# Contextual modulation of preferred social distance during the Covid-19 pandemic

**DOI:** 10.1038/s41598-021-02905-9

**Published:** 2021-12-09

**Authors:** Chiara Fini, Luca Tummolini, A. M. Borghi

**Affiliations:** 1 Department of Dynamic and Clinical Psychology and Health Studies, “Sapienza” University of Rome, Rome, Italy; 2 Institute of Cognitive Sciences and Technologies, National Research Council (CNR), Rome, Italy

**Keywords:** Human behaviour, Social behaviour

## Abstract

Social distancing during a pandemic might be influenced by different attitudes: people may decide to reduce the risk and protect themselves from viral contagion, or they can opt to maintain their habits and be more exposed to the infection. To better understand the underlying motivating attitudes, we asked participants to indicate in an online platform the interpersonal distance from different social targets with professional/social behaviors considered more or less exposed to the virus. We selected five different social targets: a cohabitant, a friend working in a hospital, a friend landed from an international flight, a friend who is back from a cycling ride, or a stranger. In order to measure the realistic and the symbolic perceived threat, we administered the Brief 10-item COVID-19 threat scale. Moreover, in order to measure the risk attitude in different domains, the participants were also asked to fill in the Domain-Specific Risk-Taking DOSPERT scale. Results reveal a general preference for an increased distance from a stranger and the friends who are considered to be more exposed to the virus: the friend working in a hospital or landed from an international flight. Moreover, the interpersonal distance from friends is influenced by the perception of Realistic Threat measured through the Integrated Covid Threat Scale and the Health/Safety Risk Perception/Assumption as measured by the DOSPERT scale. Our results show the flexible and context-dependent nature of our representation of other people: as the social categories are not unchangeable fixed entities, the bodily (e.g., spatial) attitudes towards them are an object of continuous attunement.

## Introduction

In order to contrast the transmission of the coronavirus (Sars-Cov-2) via respiratory droplets and physical contact, one of most common non-pharmaceutical interventions has been to ask citizens to maintain a social distance of at least 6 feet (1.8 m)^1^.

Such a safety threshold has been recommended by international organizations in charge of providing updated data on the pandemic progression (data updated as of 1st September 2021: 217,558,771 confirmed cases of COVID-19, including 4,517,240 deaths, https://covid19.who.int) and then of informing health authorities on the best political strategies to adopt^2,3^. Despite these official guidelines and regulations, how each person handles her or his physical distance from others is ultimately affected by personal and social influences. Each person decides the style of her/his social routines. The psychological impact of the COVID-19 crisis indeed has been unprecedented in the general population^4^ and especially in the healthcare workers around the world^5,6^. After the end of lockdown measures in many countries, resuming the pre-pandemic social habits has been probably viewed as comforting; still the anxiety associated with higher risk of contagion was also present. Affective bonds with friends have naturally led to an increased desire for connection, which is a necessary determinant of health quality^7^. However, the deep need to maintain social bonds with close family and friends has to be balanced with the risk of being potentially infected, especially when social distancing measures are violated. Hence, people have often opted for safer online interactions instead of face-to-face encounters.

The spatial distance people maintain between themselves and others can be defined as interpersonal distance^8,9^. It creates and defines the dynamics of social interactions^10^. Interpersonal distance is a salient cue of responsiveness and of a feeling of being comfortable or not: for example, short interpersonal distance can indicate emotional closeness, while being too close to strangers can create discomfort^11–14^.

According to the Threat Hypothesis^15^, people perceive threatening stimuli as spatially/temporally closer to themselves^16–18^ as compared to safe ones. Consequently, they increase their interpersonal distance to feel more comfortable again^19,20^. A reduced distance perception to threatening individuals is very functional: it might be advantageous to categorize a threat as closer to us so that behavior is subsequently undertaken to increase the distance from them^15^. Importantly, reducing interpersonal contacts and increasing interpersonal distance has been part of behavioral adaptations against epidemics^21^.

Although the safe interpersonal distance has been indicated as corresponding to approximately 2 m^1^, COVID-19 can be transferred through aerosols, meaning safe interpersonal distance should be even wider^22,23^. However, previous research has shown that people spontaneously set an interpersonal distance of about 1 m when interacting with unfamiliar others^24–26^ and this preference holds across different cultures, albeit with considerable variance^27^. Consequently, requiring people to keep additional distance from each other might involve the inhibition of emotional expressions, (i.e., social touch), (see^26^) that are important for strengthening social bonds. In a recent study, it has been demonstrated that the interpersonal distance between participants predicts the quality of the social interaction and the enjoyment level, i.e. increased interpersonal distance strongly predicts lower enjoyment level^28^. Thus, if staying farther from a friend reduces the anxiety of being infected, it may also lead to a general sense of subjective dissatisfaction.

Importantly, the literature has focused on the modulation of interpersonal distance depending on culture or the kind of out-group member one encounters^15,29–31^, gender and age^32^, moral values^33^, personality traits^34^, and emotional expressions^20^, but has neglected to investigate how our social borders can be rapidly re-shaped by an unusual health event, like a pandemic.

Here, we consider how context flexibly modulates interpersonal distance and we analyze how the current pandemic situation influences interpersonal distance evaluations overall, comparing strangers, friends in different contexts, and a cohabitant. A friend working in the hospital or a friend often flying for work are more susceptible to get infected compared with friends who can limit the potential risky situations. Another scenario pertains to social interaction with a cohabitant, a person with whom people have created a more private sphere, and with whom presumably respecting social distancing recommendations inside the home is more complicated. Finally, there are the occasional social exchanges with new people, i.e., a barman, a man on the street distributing leaflets. Social distancing from people might also be modulated by the risk attitudes (i.e., reducing the social contacts to protect the body from the virus, or maintaining the social contact habits but being more exposed to the infection). Risk-taking can be better understood in a risk-return framework, in which risk-taking is a function of the perceived risk of the action or choice option, its expected benefits, and the decision maker's attitude toward perceived risk^35^.

Our hypotheses, in keeping with the literature on interpersonal distance^19,20^, are that: (i) the preferred interpersonal distance from a social target considered as an out-group member (the stranger) is more extended, (ii) the two categories of friends perceived as more vulnerable to the infection (the friend who is working in the hospital and the friend just landed from an international flight) are kept more distant relative to the cohabitant and the neutral friend (the one back from the cycling riding). Moreover, from the administration of the brief 10-item COVID-19 threat scale^36^, we predict that the perception of Realistic threats to physical or financial safety and the perception of Symbolic threats to one's sociocultural identity differently modulate the preferred interpersonal distance from the social targets. Our hypothesis relies on evidence showing that members of an individualistic culture like the US and Italy^37,38^, who perceived high levels of Realistic Threat are more likely to adhere to social distancing even though social distancing might disrupt the norms and structures associated with their national identity. In direct contrast, the Symbolic Threat of COVID-19 to American national identity predicted less support for social distancing^36^. Finally, we explore whether individual risk attitudes in different risk domains (health/safety, ethical, financial, social, and recreational decisions measured with the Domain-Specific Risk-Taking DOSPERT scale^39^) would further differently impact on the preferred interpersonal distance from the social targets.

### Ethics statement

The study was conducted in line with the international ethical norms and approved by the ethical committee of Sapienza University (Rome, Italy), in accordance with the ethical standards of the 2013 Declaration of Helsinki. All participants gave their written informed consent to take part in the study and gave their informed consent for publication of their images/data in an online open access publication.

### Participants

The sample in Italy was collected from the second half of July to the end of August 2020. During the summer period, Italy was out of the lockdown, and most of the ordinary social activities were again permitted, although it was still recommended to respect social distancing requirements and to wear face masks. Participants were recruited via the online platform Gorilla^40^. They were required to perform an interpersonal distance task, to complete the Integrated COVID-19 Threat Scale^36^ and the Domain-Specific Risk-Taking (DOSPERT)scale^39^. Sixty Italian participants (41 F, M_age_ = 30.46, ± 8.65, range = 20–64 ) took part in the study. The size for the sample was based on an a priori power analysis to detect effects (r) greater than 0.20 with high statistical power (power = 0.80; α = 0.05, two-tailed).

## Method and materials

We exploited five avatars, and for each one, different background information was provided. Although the perceptual appearance of the avatars was kept invariant, they wore t-shirts of five different colors: yellow, green, orange, violet, blue, and each one was associated with a different profile described by five corresponding written sentences. For the first avatar, the describing sentence was: "This is one of your friends who is working in the hospital" (FRIEND-WORKING-IN-HOSPITAL), for the second one: "This is one of your friends who landed from an international flight" (FRIEND-BACK-FROM-A-TRIP), for the third one: "This is one of your friends who is just back from a bike ride" (CYCLER-FRIEND), for the fourth one: "This is a man who you never met before" (UNKNOWN PERSON), for the fifth one: "This is a man who shares the house with you" (COHABITANT). In the first part of the experiment, participants were presented with the avatar's picture located in the center of the screen and below the associated sentence. In the lower part of the screen, one of the following two questions could be read: "How close do you get? "(ACTIVE-DISTANCE) or "How close do you allow the other to get?" (PASSIVE-DISTANCE). We asked two different questions because of the well-known asymmetry effect in the estimation of interpersonal distance. Estimates might differ depending on whether the reference point for the estimation is the subject ('How close do you get?') or the others ('How close do you allow the other to get?). Due to the effect of a self-centering schema, participants feel that others occupy their own space more than they occupy others' space^41^.

Participants could prefer to keep an interpersonal distance from the social targets, ranging from 60 cm to 8 m. It can be argued that participants understood the aim of the experiment and responded in line with what the experimenters expected (demand effect). Several factors suggest that this might not be the case. First, the experiment was run online, and this renders it less likely that the participants conformed to what was expected by an experimenter that is physically absent. Second, most of the social categories we used are ambiguous, and it is unclear what sort of answer is expected. Finally, we asked two questions, one pertaining to the active and one the passive distance. The presence of these two questions might act as an additional control on the delivered responses. We have decided for 60 cm as an inferior spatial limit on the basis of the cross-cultural proxemic studies which indicate that preferred interpersonal distance ranges from 77 cm (Argentina) to 140 cm (Romania), Germany and the United States being in the middle with values of 96 and 95 cm, respectively^27^. The superior limit of 8 m corresponds to the Near extrapersonal space^42^, which is defined as a multisensorial, body-scaled space, where we walk and navigate. Beyond that threshold, the space is defined as distant, far^43^.

**Figure 1:**
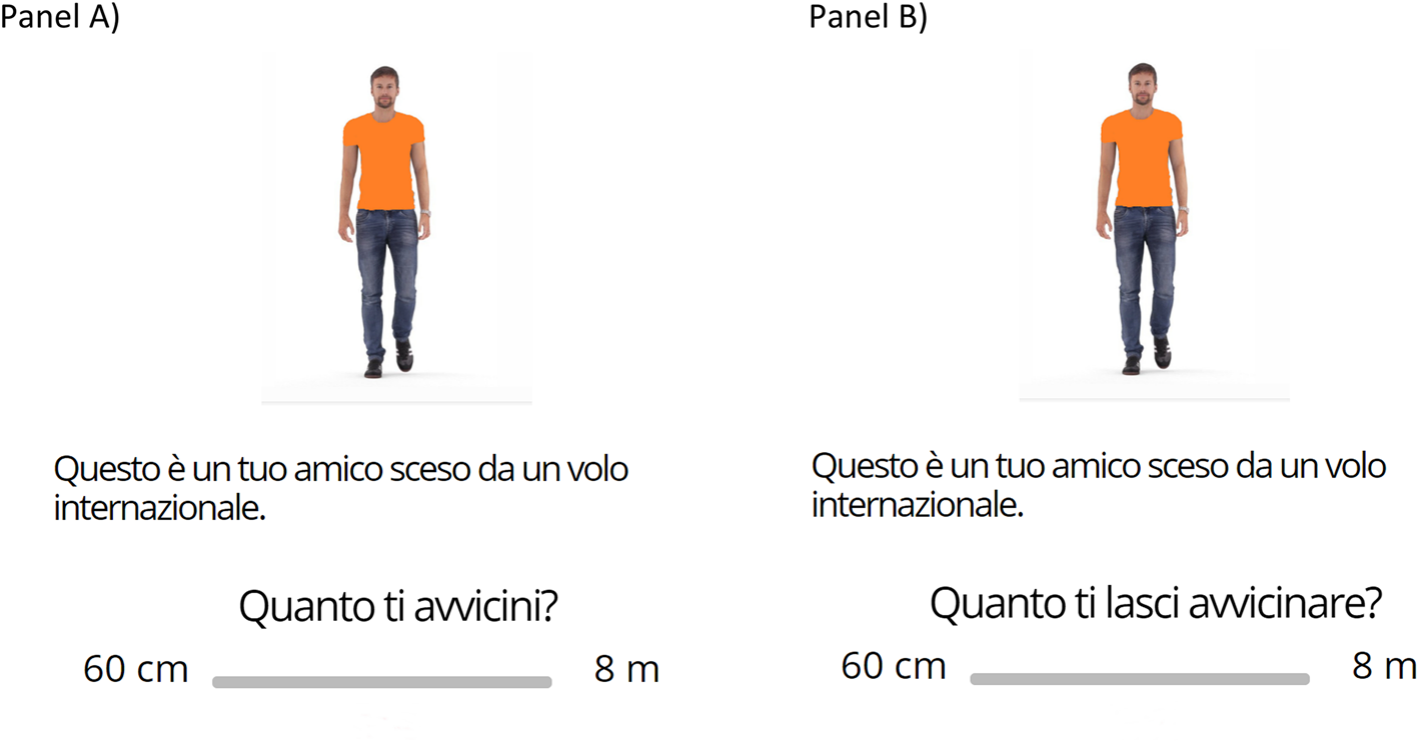
(**A**) Example of the “Active” physical-distancing practice from the avatar through a visual analog 60 cm–8 m scale (VAS); (**B**) Example of the “Passive” physical-distancing practice from the avatar through a visual analog 60 cm–8 m scale (VAS).

We administered a randomized sequence of 30 trials, which were counterbalanced in the avatar conditions (6 FRIEND-WORKING-IN-HOSPITAL trials, 6 FRIEND-BACK-FROM-A-TRIP trials, 6 CYCLER-FRIEND trials, 6 UNKNOWN PERSON trials, 6 COHABITANT trials) and the Question type (3 ACTIVE-DISTANCE, 3 PASSIVE-DISTANCE questions for each avatar condition). Four different T-shirts color combinations were created and counterbalanced across participants. Participants had to indicate their physical-distancing practice from the avatar through a visual analog 60 cm–8 m scale (VAS), (Fig. 1). When participants finished the first part of the experiment, they were asked to complete two different scales translated into Italian: the Integrated COVID-19 Threat Scale^36^ (Table 1) and the Domain-Specific Risk-Taking (DOSPERT) scale^39^ (Table 2). The first one was a validated brief 10-item COVID-19 threat scale that assesses (i) Realistic threats to physical or financial safety (first five items), and (ii) Symbolic threats to one's sociocultural identity (second five items). The Integrated COVID-19 Threat Scale evaluates how threatening the pandemic is, for each healthy, social, financial aspect, on a 4-point rating scale ranging from 1 (Not at all threatening) to 4 (Strongly threatening). Ratings are added across all items of the two subscales to obtain subscale scores, with higher scores indicating perceptions of greater threat in the Realistic Threat or Symbolic Threat subscales. The Realistic threat is the danger to the physical health and financial wellbeing of both individuals and their group, while the Symbolic Threat is the danger to group values and identity^36^. The two kinds of threat diverge in their relationship to restrictive public health behaviors: Realistic threat predicted greater self-reported adherence, whereas Symbolic Threat predicted less self-reported adherence to social-disconnection behaviors^36^.

**Table 1:** The integrated COVID-19^36^ threat scale adapted to Italy.

	Not a threat	Minor threat	Moderate threat	Major threat
Your personal health				
The health of the Italian population at whole				
Your personal financial safety				
The Italian economy				
Day to day life in your local community				
The right and freedom of the Italian population at whole				
What it means to be Italians				
Italian values and traditions				
Italian democracy				
The maintenance of law and order in Italy				

Indicate how threatened you feel by the impact of COVID 19 on the aspects listed.

**Table 2:** The Domain-Specific Risk-Taking (DOSPERT) scale^39^.

Risk perception						
1	2	3	4	5	6	7
Not at all risky	Slightly risky	Somewhat risky	Moderately risky	risky	Very risky	Extremely risky

Risk assumption						
1	2	3	4	5	6	7
Extremely unlikely	Moderately unlikely	Somewhat unlikely	Not sure	Somewhat likely	Moderately likely	Extremely likely
1. Admitting that your tastes are different from those of a friend. (S)						
2. Going camping in the wilderness. (R)						
3. Betting a day’s income at the horse races. (F)						
4. Investing 10% of your annual income in a moderate growth mutual fund. (F)						
5. Drinking heavily at a social function. (H/S)						
6.Taking some questionable deductions on your income tax return. (E)						
7. Disagreeing with an authority figure on a major issue. (S)						
8. Betting a day’s income at a high-stake poker game. (F)						
9. Having an affair with a married man/woman. (E)						
10. Passing off somebody else’s work as your own. (E)						
11. Going down a ski run that is beyond your ability. (R)						
12. Investing 5% of your annual income in a very speculative stock. (F)						
13. Going whitewater rafting at high water in the spring. (R)						
14. Betting a day’s income on the outcome of a sporting event. (F)						
15. Engaging in unprotected sex. (H/S)						
16. Revealing a friend’s secret to someone else. (E)						
17. Driving a car without wearing a seat belt. (H/S)						
18. Investing 10% of your annual income in a new business venture. (F)						
19. Taking a skydiving class. (R)						
20. Riding a motorcycle without a helmet. (H/S)						
21. Choosing a career that you truly enjoy over a more secure one.(S)						
22. Speaking your mind about an unpopular issue in a meeting at work. (S)						
23. Sunbathing without sunscreen. (H/S)						
24. Bungee jumping off a tall bridge. (R)						
25. Piloting a small plane. (R)						
26. Walking home alone at night in an unsafe area of town. (H/S)						
27. Moving to a city far away from your extended family. (S)						
28. Starting a new career in your mid-thirties. (S)						
29. Leaving your young children alone at home while running an errand. (E)						
30. Not returning a wallet you found that contains $200. (E)						

For each of the following statements, please indicate the likelihood that you would engage in the described activity or behavior if you were to find yourself in that situation. Provide a rating from Extremely Risky/Unlikely to Extremely Risky/Likely, using the above scale.

E = Ethical, F = Financial, H/S = Health/Safety, R = Recreational, and S = Social.

The Domain-Specific Risk-Taking (DOSPERT) scale^39^ assesses either conventional risk attitudes (defined as the reported level of risk-taking) and perceived-risk attitudes (defined as the willingness to engage in a risky activity as a function of its perceived riskiness) in five commonly encountered content domains, i.e., ethical, financial (further decomposed into gambling and investment), health/safety, social, and recreational decisions^39^. Crucially, how much an individual's judged level of perceived risk (risk perception) decreases his (her) likelihood of engaging in risky behaviors across domains (risk assumption). The impact of risk perception on risk assumption is the perceived-risk attitude, and according to the risk-return model of risky choice, it is a stable individual characteristic^39^. The Domain-Specific Risk-Taking (DOSPERT) scale evaluates the assessment of how risky each behavior is on a 7-point rating scale ranging from 1 (Not at all) to 7 (Extremely Risky). Ratings are added across all subscale items to obtain subscale scores, with higher scores suggesting perceptions of greater risk in the subscale domain.

### Data analysis

The analysis included 1800 observations on the preferred interpersonal distance thresholds (6 trials: 3 active-distance, 3 passive-distance) trials for the 5 contexts FRIEND-WORKING-IN-HOSPITAL, FRIEND-BACK-FROM-A-TRIP, CYCLER-FRIEND, UNKNOWN PERSON, COHABITANT measured in 60 participants). We performed multivariate mixed models with R Studio software (R packages lme4, lsmeans, lmerTest, ggplot2, ggthemes, afex, nlme—version 3.6.3). Following a stepwise procedure in the first model, we included fixed effects for the variables Context (FRIEND-WORKING-IN-HOSPITAL, FRIEND-BACK-FROM-A-TRIP, CYCLER-FRIEND, UNKNOWN PERSON, COHABITANT), Type of Question (ACTIVE-DISTANCE, PASSIVE-DISTANCE), and the interaction between the two categorical predictors. Participants and color combinations were entered as random intercepts. In the second model, we excluded the Context × Type of Question interaction, and in the third model, we excluded the fixed effect for the variable Type of Question. In all three models, we constantly kept the random intercepts for participants and color combinations. The third model best fitted the data distribution, see Table 3). We observed no severe violation of the homoscedasticity or normality assumptions at a visual inspection of the residual plots. Statistical significance of fixed effects was determined using type III ANOVA test (the p-values for the fixed effects were calculated from an F test on Sattethwaite's approximation), with the mixed-function from afex package. We performed post-hoc comparisons with the 'Estimated Marginal Means' R package (version 1.3.3^44^) via the emmeans function and Tukey correction for multiple comparisons.

**Table 3:** (AIC) Akaike's information criteria, (BIC) Schwarz's Bayesian criterion, (− 2LL) − 2 LL-Likelihood.

		AIC	BIC	− 2LL
Model 1	Fixed factors: context, distance question, context × distance question interaction	6590.7	6662.1	− 3282.4
	Random factors: subjects, color combinations			
Model 2	Fixed factors: context, distance question	6583.6	6633.1	− 3282.8
	Random factors: subjects, color combinations			
Model 3	Fixed factors: context	6582.3	6626.3	− 3283.2
	Random factors: subjects, color combinations			

Information criteria on the linear mixed models performed on the entire sample.

## Results

**Figure 2:**
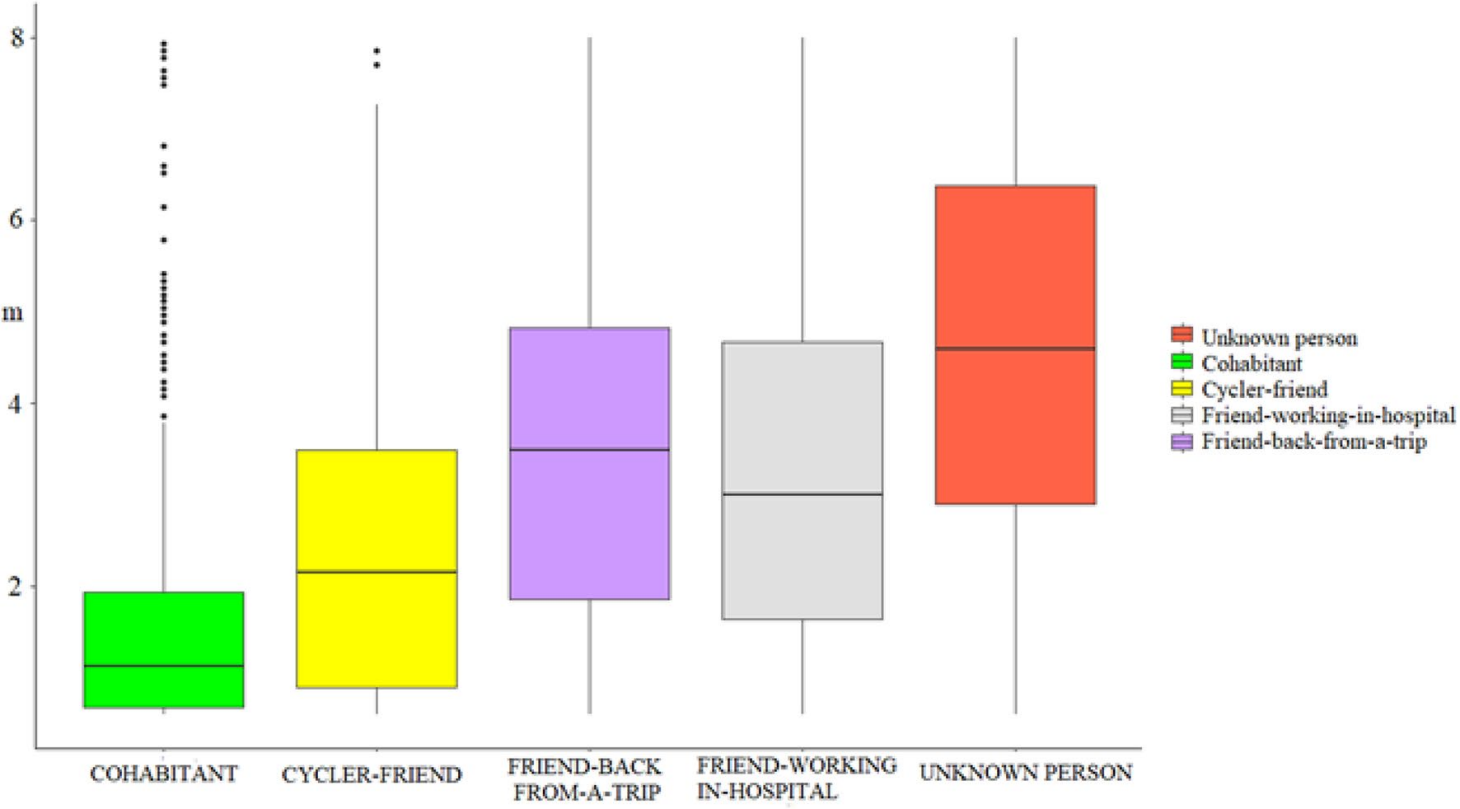
The graph shows the predicted values of the third model, which is the one with the best fitting. The analysis yielded a significant main effect of the Context (F(4,1735) = 226.51, p = 0.001). The results show that the preferred interpersonal distance threshold is more extended with the UNKNOWN PERSON than with all the other contexts: COHABITANT, which was the most reduced one compared with the CYCLER-FRIEND, the FRIEND-WORKING-IN-HOSPITAL and the FRIEND-BACK-FROM-A-TRIP. All these measures significantly differed from each other, p<0.00001. Only the preferred interpersonal distance with the FRIEND-WORKING-IN-HOSPITAL was not significantly different from the preferred interpersonal distance with the FRIEND-BACK-FROM-A-TRIP, p = .1009, Horizontal lines in the boxes indicate the median, upper and lower borders indicate I and III quartile, "whiskers" extend to the farthest points that are not outliers, dots represent outlier trials.

The third model [R2c = 0.58]^45^, yielded a significant main effect of Context (F(4,1735) = 226.51, p=0.0001). Tukey post hoc comparisons showed that the preferred interpersonal distance threshold was more extended with the UNKNOWN PERSON (4.64 m, SE=0.214) than with all the other contexts: COHABITANT, which was the most reduced one (1.72 m, SE=0.214), CYCLER-FRIEND (2.40 m, SE=0.198), FRIEND-WORKING-IN-HOSPITAL (3.36 m, SE=0.214), and FRIEND-BACK-FROM-A-TRIP (3.62 m, SE=0.214). All these measures significantly differed from each other, p<0.00001. Only the preferred interpersonal distance with the FRIEND-WORKING-IN-HOSPITAL was not significantly different from the preferred interpersonal distance with the FRIEND-BACK-FROM-A-TRIP, p=0.1009, Fig. 2). These results suggest that participants prefer to keep a more extended distance from people with whom they have never interacted before and towards those with whom they do not have any affective bond. In contrast, the person with whom they share the domestic space is kept at the closest distance; probably living in the same house induces the people to develop more intimacy. It is then interesting to notice that both the friend who is working in the hospital and the friend just back from the airport are kept equally far compared with the friend back from the cycling ride**.** The friends' categories considered at risk to get infected by the virus seem to induce the adoption of a more robust social distancing compared with a friend's category who does not have a risky social and professional profile.

### Integrated covid threat scale and interpersonal distance

**Figure 3:**
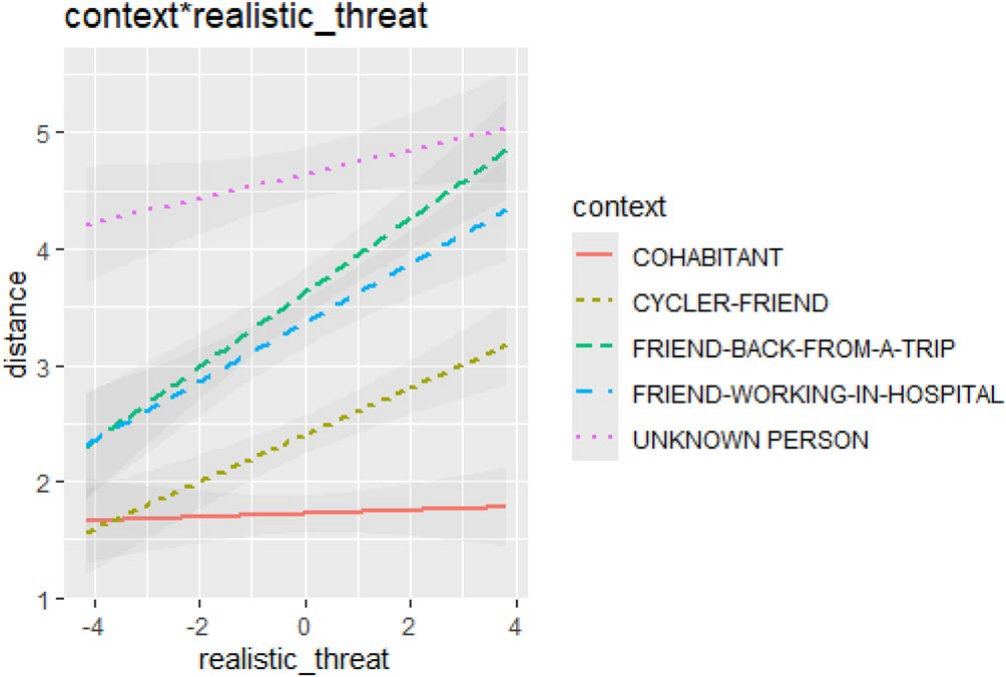
The graph shows the predicted values of the outcome variables. Shaded bands represent the confidence intervals (95%). The more participants showed high scores of Realistic Threat, the more they decided to keep a distance from the three categories of friends.

To test a possible association between the threat posed by COVID 19 and interpersonal distance thresholds, we added the Symbolic and Realistic threat subscales of the Integrated Covid Threat Scale as continuous predictor and as categorical predictors the Context (FRIEND-WORKING-IN-HOSPITAL, FRIEND-BACK-FROM-A-TRIP, CYCLER-FRIEND, UNKNOWN PERSON, COHABITANT). Participants and color combinations were kept as random intercepts. We observed no severe violation of the homoscedasticity or normality assumptions at a visual inspection of the residual plots. The model [R2c = 0.59]^45^, yielded a significant main effect of the Context (F(4,1727.005) = 231.8041, p < 0.00001) and of the continuous predictor Realistic Threat (F(1,57.002) = 4.4605, p=0.03908), but not of the Symbolic Threat (F(1,57.000) = 0.2753, p = 0.60185). We found a significant two-way interaction between the Context and the continuous predictor Realistic Threat (F(4,1727.005) = 11.2147, p < 0.00001). We performed estimates of slopes of the covariate trend for each level of the factor with the 'Estimated Marginal Means' R package (version 1.3.3^44^) via the emtrends functions. Simple slope analysis showed that the slopes of the FRIEND-WORKING-IN-HOSPITAL [LCI 0.0683–UCI 0.429], FRIEND-BACK-FROM-A-TRIP [LCI 0.1314–UCI 0.492], CYCLER-FRIEND [LCI 0.0104–UCI 0.371] were significantly different from zero as a function of the Realistic Threat. The pairwise difference between the simple slopes of FRIEND-BACK-FROM-A-TRIP and COHABITANT as a function of the Realistic Threat was significant (estimate = 0.2996, SE = 0.0506, t(1727) = 5.918 p < 0.0001). The pairwise difference between the simple slopes of the FRIEND-BACK-FROM-A-TRIP and the UNKNOWN PERSON as a function of the Realistic Threat (estimate = 0.2185, SE = 0.0506, t(1727) = 4.313 p = 0.0002), as the pairwise difference between the CYCLER-FRIEND and the COHABITANT (estimate = 0.1786, SE = 0.0506, t(1727) = 3.529 p = 0.0039) were significant. Also, the pairwise difference between the simple slopes of the FRIEND-WORKING-IN-HOSPITAL and the COHABITANT was significant (estimate = 0.2364, SE = 0.0506, t(1727) = 4.671 p < 0.0001) and, finally the pairwise difference between the simple slopes of the FRIEND-WORKING-IN-HOSPITAL and the UNKNOWN PERSON (estimate = 0.1551, SE = 0.0506, t(1727) = 3.066 p = 0.0187), Fig. 3). The more participants showed high scores of Realistic Threat, the more they decided to keep a distance from the three categories of friends, in line with the fact that the Realistic Threat predicts a more pronounced adherence to socially restrictive public health behaviors like social distancing^36^.

### Health/safety risk perception and assumptionof the domain-specific risk-taking (DOSPERT) and interpersonal distance

**Figure 4:**
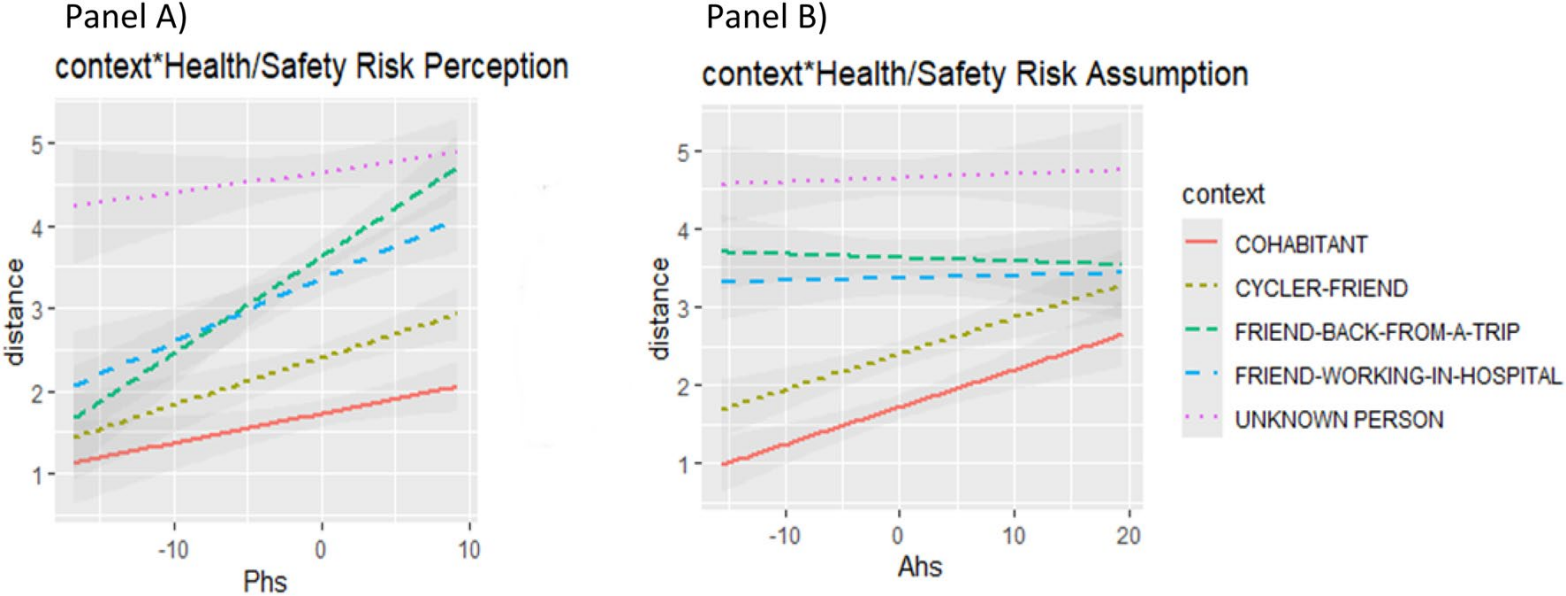
(**A**) The graph shows the predicted values of the outcome. Shaded bands represent the confidence intervals (95%). The higher participants’ scores of Health/Safety Risk Perception are, the more they decided to keep a distance from the three categories of friends. (**B**) The graph shows the predicted values of the outcome variables. Shaded bands represent the confidence intervals (95%). Results indicate that the higher participants’ score in the Health/Safety Risk Assumption are, the more they keep distance from the Cohabitant and Cycler Friend.

The analysis on the ethical, social, financial and recreational Risk Perception and Assumption scales are reported in the supplementary materials. We reported here the analysis including as continuous predictors the Health/Safety Risk Perception and Assumption of the Domain-Specific Risk-Taking (DOSPERT) scale and as categorical predictors the Context (FRIEND-WORKING-IN-HOSPITAL, FRIEND-BACK-FROM-A-TRIP, CYCLER-FRIEND, UNKNOWN PERSON, COHABITANT), participants and color combinations were kept as random intercepts. We observed no severe violation of the homoscedasticity or normality assumptions at a visual inspection of the residual plots. The model [R2c = 0.60]^45^ yielded a significant main effect of the Context, (F(4,1727.005) = 232.9861, p<0.00001) and of the continuous predictor Health/Safety Risk Perception (F(1,57.768) = 5.5234, p = 0.02226). The two-way interaction between the continuous predictor Health/Safety Risk Perception and the categorical predictor Context was significant (F(4,1727.003) = 7.1772, p<0.00001) as the two-way interaction between the continuous predictor Health/Safety Risk Assumption and the categorical predictor Context (F(4,1727.004) = 6.1742, p<0.00001). Simple slope analysis showed that the slopes of the FRIEND-WORKING-IN-HOSPITAL [LCI 0.01524–UCI 0.1439], FRIEND-BACK-FROM-A-TRIP [LCI 0.05441–UCI 0.1831], CYCLER-FRIEND [LCI 0.00791–UCI 0.1367] were significantly different from zero as a function of the Health/Safety Risk Perception. The pairwise difference between the simple slopes of FRIEND-BACK-FROM-A-TRIP and COHABITANT as a function of Health/Safety Risk Perception (estimate = 0.06895, SE = 0.0186, t(1727) = 3.708 p=0.0020), as the pairwise difference between the simple slopes of the FRIEND-BACK-FROM-A-TRIP and the UNKNOWN PERSON (estimate = 0.09444, SE = 0.0186, t(1727) = 5.079 p<0.0001) were significant. The pairwise difference between the simple slopes of the FRIEND-WORKING-IN-HOSPITAL and the UNKNOWN PERSON as a function of Health/Safety Risk Perception was significant (estimate = 0.05527, SE = 0.0186, t(1727) = 2.972 p=0.0249) Fig. 4). The higher the level of Health/Safety Risk Perception was, the more participants decided to keep their distance from the three categories of friends. This pattern of results overlaps with the one found for the Realistic Threat subscale of the Integrated COVID-19 Threat Scale. Experiencing the risk that our health is vulnerable to the infection impacts on the preferred interpersonal distance which participants decide to keep from friends and not from the cohabitant or the unknown person.

Simple slope analysis showed that the slopes of the CYCLER-FRIEND [LCI 0.0179–UCI 0.1181] and the COHABITANT [LCI 0.0162–UCI 0.1164] were significantly different from zero as a function of the Health/Safety Risk Assumption. The pairwise difference between the simple slopes of the FRIEND-BACK-FROM-A-TRIP and the CYCLER-FRIEND as a function of Health/Safety Risk Assumption (estimate = − 0.0423, SE = 0.0135, t(1727) = − 3.131 p=0.0153), as the pairwise difference between the simple slopes of the FRIEND-BACK-FROM-A-TRIP and the COHABITANT (estimate = − 0.04065, SE = 0.0135, t(1727) = − 3.004 p=0.0227) were significant. The pairwise difference between the simple slopes of the CYCLER-FRIEND and the FRIEND-WORKING-IN-HOSPITAL as a function of Health/Safety Risk Assumption (estimate = 0.04095, SE = 0.0135, t(1727) = 3.026 p=0.0212) and the pairwise difference between the simple slopes of the CYCLER-FRIEND and the UNKNOWN PERSON (estimate = 0.04856, SE = 0.0135, t(1727) = 3.584 p=0.0032) were significant. Finally, both the pairwise difference between the simple slopes of the FRIEND-WORKING-IN-HOSPITAL and the COHABITANT (estimate = − 0.03924, SE = 0.0135, t(1727) = − 2.900 p=0.0309) and the pairwise difference between the simple slopes of the COHABITANT and the UNKNOWN PERSON (estimate = 0.04682, SE = 0.0135, t(1727) = 3.458 p=0.0050) were significant, Fig. 4). These results indicate that the higher their scores are in the Health/Safety Risk Assumption, the more participants keep distance from the cohabitant and cycler friend. It is interesting to highlight that the social targets considered less controllable/more dangerous (friend working in the hospital and friend back from a trip, unknown person) are not impacted by the Health/Safety Risk Assumption on the preferred interpersonal distance; participants decide to assume the risk of being closer only to the social categories considered “safe” such as the cycler friend, and “controllable” such as the cohabitant.

## Discussion

In this study, we have investigated the preferred spatial distance from different social targets from the second half of July to the end of August 2020. At that time, the extreme isolation restrictions enacted in Italy were lifted, the contagion rate dropped down, and people were facing an ambiguous scenario where social behaviors were regulated mainly by the individual sense of responsibility. Indoor and outdoor social distancing was mandatory, but each individual adjusted the physical closeness to relatives, friends, and others within the private domain. The question we addressed here is whether people adjust the interpersonal distance from different social categories with respect to the affective bond and the social/professional profiles, which can be perceived as more or less vulnerable to contagion. We selected the following targets: a friend just got off from an international flight, a friend working in a hospital, a friend just back from a cycling ride, an unknown person, and a cohabitant. In line with our hypotheses, we have shown that (1) people kept a more extended interpersonal distance from the unknown person compared with the others, that (2) the two friends more exposed to the infection (a friend just got off from an international flight, a friend who is working in a hospital), were kept equally far from the neutral one (the cycling friend) and that (3) the cohabitant was the one from whom people stayed closer.

The less we know about someone, as in the case of a person we never met before, the more we feel suspicious, and we opt to adopt protective behaviors. From an evolutionary perspective, it is usually adaptive for organisms to respond to potential threats as if they were truly threatening rather than to fail to respond^46^. In keeping with the Threat Hypothesis^15^, people perceive a threatening stimulus as spatially closer to themselves^16–18^ as compared to a positive one, and consequently, they increase the interpersonal space to reinstall a comfortable distance^19,20^. The evolutionary values of these chosen interpersonal distances clearly indicate the need to adopt protective behaviors besides the need to maintain satisfactory interactions which are strongly predicted by a reduced interpersonal distance^28^.

One psychological challenge imposed by the pandemic concerns the emotional, cognitive and behavioral conflict on the social human needs: the necessity to experience a feeling of affiliation to deal with a dramatic world crisis, which paradoxically implies to look at ourselves and the others as potentially reciprocally harmful, and consequently the urgency to restructure the behavioral, emotional expressions towards the social network. In the pandemic context, the awareness that our own and the other's body can be the vehicle of the virus leads to developing a general sense of alertness, which is oriented towards all our social contacts and amplified by the assessment of their social behaviors. In keeping with this, at least in part, the psychological distance does not overlap anymore with the physical distance^47^. Two friends more susceptible to get the infection, i.e., a friend just got off from an international flight or a friend working in the hospital, are kept at a farther distance compared with a friend just back from a cycling ride. Such result expresses the impact of the COVID-19 perceived threat, which partially overcomes the affective behavioral expressions associated with the other's social identity, and rather imposes a prompt attentional focus on the "bodies" as potentially dangerous, at the expense of the focus on the idiosyncratic relationship with the individual. This speculation can explain the extended interpersonal distance towards the two categories of friends being perceived as targets more exposed to the contagious. The results also indicate that a person with whom we share the house, a cohabitant, is kept at the closest interpersonal distance than the other social categories. Indeed, regardless of the emotional relationship with her/him, sharing a concrete private context already sets some spatial constraints. This might pose a lighter charge of responsibility in respecting the social distancing.

It is worth specifying that since the study was performed on-line, not in a real setting or in an immersive virtual reality setup, it did not aim at extracting absolute values of interpersonal distance estimation but rather at exploring the relation among the depicted contexts.

Despite these limitations, our results indicate that the higher people scored on the Realistic but not on the Symbolic Threat subscale of the brief 10-item COVID-19 threat scale^36^, the more they kept a distance from the three categories of friends. The COVID-19 disease poses real threats to an individual's or group's physical health and economic wellbeing and to the integrity or validity of a group's meaning system^48–52^. Evidence collected on an American sample testifies that Americans who perceived high levels of Realistic Threat were more likely to adhere to social distancing even though social distancing might disrupt the norms and structures they might associate with American identity. In direct contrast, the Symbolic Threat of COVID-19 to American national identity predicted less support for social distancing^36^. Accordingly, our results provide additional information: the Realistic Threat modulates the interpersonal distance from the friends. With them, we experience the ambivalent need to be closer and sufficiently distant at the same time. It can be speculated that the preferred interpersonal distance from friends, compared with the other two targets (the cohabitant or the unknown person), might require a stronger sense of responsibility because of the affective conflict: on the one side, the distress and the anxiety to be infected^36^, on the other side the need to conserve the emotional bond with meaningful people^7^. Such affective conflict would not be present with the cohabitant because sharing the house is a social condition that cannot be avoided, and it would also not be present with the unknown person since any kind of social interaction can be easily avoided. An equal pattern of results is found with the healthy/safety perception subscale of the Domain-Specific Risk-Taking(DOSPERT)^39^. The healthy/safety subscale evaluates the respondents' level assessment of how risky each behavior is for health decisions (e.g., seat usage, smoking). Respecting the social distancing from friends is a behavior fueled by the psychological motivation to protect not only ourselves from others but also to protect them from us, especially those other people towards whom we feel more responsible, as the loved ones. The more people care about the healthy/safety dimension, the more they are willing to pay the personal cost of staying far from valued close people^53^. Finally, the higher people scored in the Health/Safety Risk Assumption, the more they kept their distance from the cohabitant and cycler friend. As already speculated, participants might decide to assume the risk of being closer only to the social categories considered “safe” such as the cycler friend, and “controllable” such as the cohabitant. The other three social targets were not impacted by the risk assumption, because they were modulated by the risk perception, which might inhibit approaching behaviors towards them. Importantly, our results draw attention to the social vulnerability of specific social categories during the pandemic: keeping more distance from who is working in a hospital and who travel outside the country, might be the expressions of discriminatory behaviors largely documented towards specific ethnic groups^54^ or/and healthcare workers perceived as more contagious, who experience avoidance by their family/friends or community owing to stigma or fear^55,56^.

## Conclusion

The novelty of our study consists of showing how the current context might influence the perception of interpersonal distance. Previous work focused on the influence of cultures on interpersonal distance or on how people perceived as potentially risky, e.g., outgroup members, influence our estimations. Here we found that the spread of COVID-19 impacts how we perceive other people, from unknown people to cohabitants. Even though the same study was not conducted before and after the spread of the pandemics, the pattern of results and the comparison with previous data in other cultures strongly suggest that the results were modulated by the current context. Crucially, we found that the current pandemic context modulates how we perceive friends. We update our representation of friends as a function of what they’ve done (working in a hospital, traveling abroad). The results thus underline the flexible and context-dependent aspects in our representation of other people. Importantly, our study also pointed out an important role that emotions can play in updating our conceptual representation of others. Understanding the relationship between the preferred physical distance from specific social categories (friends or not) and how specific risk traits or Covid-related risk regulate the social distancing can help to identify the contexts where the restriction adherence may produce more or less psychological fatigue.

## Supplementary Information

Supplementary Information.

## Data Availability

Data available at https://osf.io/fj53d/.
