# Supplementary material for: Contextual modulation of preferred social distance during the Covid-19 pandemic
Source: Sci Rep. 2021 Dec 9;11:23726. doi: 10.1038/s41598-021-02905-9 (PMC8660879; doi:10.1038/s41598-021-02905-9)
Supplement: Supplementary file 1 — Supplementary Information. [file 41598_2021_2905_MOESM1_ESM.pdf]

Supplementary Materials

Protect me from my friends: Representing the distance from others during the Covid-19 pandemic in Italy

Chiara Fini[1] , Luca Tummolini[2] , A.M. Borghi[1,2]

[1]Department of Dynamic and Clinical Psychology and Health Studies, "Sapienza" University of Rome, Italy

[2]Institute of Cognitive Sciences and Technologies, National Research Council (CNR), Rome, Italy

Social risk Perception and Assumption of the Domain-Specific Risk-Taking (DOSPERT) and Interpersonal distance

A further analysis was performed to test the Impact of the Social Risk Perception and Assumption of the Domain-Specific Risk-Taking (DOSPERT) subscales on the preferred interpersonal distance thresholds in each social context. The model included as continuous predictors the Social Risk Perception and Assumption of the Domain-Specific Risk-Taking (DOSPERT) scale and as categorical predictors the Context (FRIEND-WORKING-IN-HOSPITAL, FRIEND-BACK-FROM-A-TRIP, CYCLER-FRIEND, UNKNOWN PERSON, COHABITANT), participants and color combinations were kept as random intercepts. The model yielded a significant main effect of the Context (FRIEND-WORKING-IN-HOSPITAL, FRIEND-BACK-FROM-A-TRIP, CYCLER-FRIEND, UNKNOWN PERSON, COHABITANT), ($F(4,1727.005)=239.6348$, $p<.00001$) and two-way interactions between the Context, the Social Risk Perception ($F(4,1727.003)=21.3699$, $p<00001$) and the Social Risk Assumption ($F(4,1727.003)=5.0360$, $p=0.0005$) were significant. Simple slope analysis showed that the slopes of the FRIEND-BACK-FROM-A-TRIP [LCI 0.06637- UCI 0.1839] and the UNKNOWN PERSON [LCI 0.00453- UCI 0.1221] were significantly different from zero as a function of the Social Risk Perception. The pairwise difference between the simple slopes of the FRIEND-BACK-FROM-A-TRIP and the CYCLER-FRIEND as a function of the Social Risk Perception was significant (estimate=0.1116, SE = 0.016, $t(1727)=6.986$ $p<.0001$), as the pairwise difference between the simple slopes of the FRIEND-BACK-FROM-A-TRIP and the FRIEND-WORKING-IN-HOSPITAL (estimate=0.0829, SE =0.016, $t(1727)=5.192$ $p<.0001$). The pairwise difference between the simple slopes of the FRIEND-BACK-FROM-A-TRIP and the COHABITANT (estimate =0.1364, SE=0.016, $t(1727)=8.539$ $p<.0001$) and the pairwise difference between the simple slopes of the FRIEND-BACK-FROM-A-TRIP and the UNKNOWN PERSON (estimate=0.0618, SE=0.016, $t(1727)=3.871$ $p=.0011$) were significant. The pairwise difference between the simple slopes of the CYCLER-FRIEND and the UNKNOWN PERSON (estimate=-0.0497, SE =0.016, $t(1727)=-3.114$ $p=.0161$) was significant. Finally, the pairwise difference between the simple slopes of the FRIEND-WORKING-IN-HOSPITAL and the COHABITANT (estimate=0.0534, SE =0.016, $t(1727)=3.347$ $p=.0074$) and the pairwise difference between the simple slopes of the COHABITANT and the UNKNOWN PERSON (estimate=-0.0746, SE= 0.016, $t(1727)=-4.667$ $p<.0001$) were significant Figure)5). The results suggest that the social risk perception subscale impacts the two social targets, which can be classified as out-group members: a person with whom participants have never interacted before and a person who has just been outside the country. The more participants showed higher social risk perception, the more they kept a distance from the "out-group members". Simple slope analysis showed that the slopes of the five levels of the Social Context were not significantly different from zero as a function of the Social Risk Assumption.

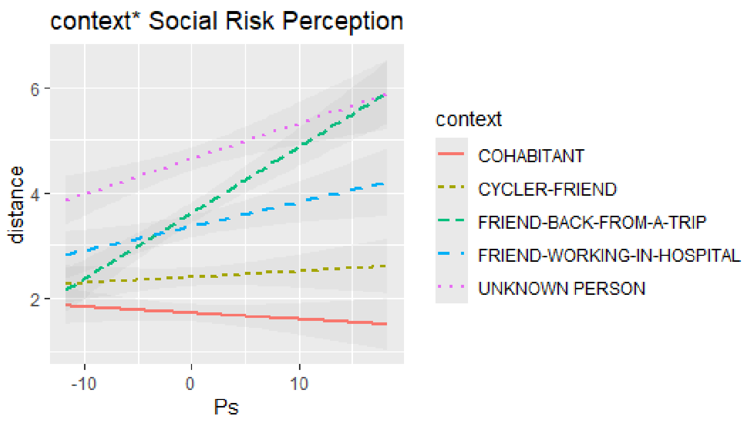

Supplementary-Figure 5) The graph shows the predicted values of the outcome variables. Shaded bands represent the confidence intervals (95%). The more participants showed high scores of Social Risk perception, the more they decided to keep a distance from the Unknown person and the Friend back from a trip.

Ethical Risk Perception and Assumption of the Domain-Specific Risk-Taking (DOSPERT) and Interpersonal distance

The analysis included as continuous predictors the Ethical Risk Perception and Assumption of the Domain-Specific Risk-Taking (DOSPERT) scale and as categorical predictors the Context (FRIEND-WORKING-IN-HOSPITAL, FRIEND-BACK-FROM-A-TRIP, CYCLER-FRIEND, UNKNOWN PERSON, COHABITANT). Participants and color combinations were kept as random intercepts. The model yielded a significant main effect of the Context (FRIEND-WORKING-IN-HOSPITAL, FRIEND-BACK-FROM-A-TRIP, CYCLER-FRIEND, UNKNOWN PERSON, COHABITANT), (F(4,1728.006)=233.1498, p<.00001) and of the continuous predictor Ethical Risk Perception (F(1,56.904)=9.1407, p=.003745). The two-way interaction between the continuous predictor Ethical Risk Perception and the categorical predictor Context was significant (F(4,1727.003)=12.4037, p<.00001). Simple slope analysis showed that the slopes of the FRIEND-WORKING-IN-HOSPITAL [LCI 0.04807-UCI 0.1563], FRIEND-BACK-FROM-A-TRIP [LCI 0.06738- UCI 0.1756], CYCLER-FRIEND [LCI 0.01687- UCI 0.1251] were significantly different from zero as a function of the Ethical Risk Perception. The pairwise difference between the simple slopes of the FRIEND-BACK-FROM-A-TRIP and the CYCLER-FRIEND as a function of the Ethical Risk Perception (estimate=0.0505, SE =0.0159, t(1727)=3.179 p=.0130) and the pairwise difference between the simple slopes of the FRIEND-BACK-FROM-A-TRIP and the COHABITANT as a function of Ethical Risk Perception were significant (estimate = 0.0991, SE=0.0159, t(1727)=6.236 p<.0001). The pairwise difference between the simple slopes of the FRIEND-BACK-FROM-A-TRIP and the UNKNOWN PERSON as a function of Ethical Risk Perception (estimate= 0.0708, SE=0.0159, t(1727)=4.456 p=.0001) and the pairwise difference between the simple slopes of the CYCLER-FRIEND and the COHABITANT (estimate = 0.0486, SE =0.0159, t(1727)=3.057 p=.0192) were significant. The pairwise difference between the simple slopes of the FRIEND-WORKING-IN-HOSPITAL and the COHABITANT as a function of Ethical Risk Perception was significant

(estimate = 0.0798, SE =0.0159, t(1727)=5.021 p<.0001) and the pairwise difference between the simple slopes of the FRIEND-WORKING-IN-HOSPITAL and the UNKNOWN PERSON (estimate = 0.0515, SE =0.0159, t(1727)=3.241 p=.0106) were significant, Fig 6). Again, as for the covariates Realistic Threat and Healthy/Safety Risk perception, also the Ethical Risk perception impact on the three categories of friends but not on the unknown person or the cohabitant. The results suggest that being in a friendly relation with someone involves two kinds of conflicts: the emotional conflict between the need to preserve the emotional bond and the need to protect ourselves from the contagion together with the ethical conflict between the need to maintain the emotional bond and the responsibility towards the community in respecting the social norms. There is instead no impact of Ethical Risk perception with the cohabitant or the unknown person because they are less susceptible, as previously remarked, to induce an emotional conflict that undermines the respecting of social distancing.

Simple slope analysis showed that the slope of the CYCLER-FRIEND [LCI 0.00744- UCI 0.1085] was significantly different from zero as a function of the Ethical Risk Assumption, Figure 6). Only the preferred interpersonal distance from the cycler friend was impacted by the Ethical Risk Assumption. It can be speculated that the other two friends are perceived as dangerous, like the unknown person, compared with the cycler friend (the neutral one), while the cohabitant might be conceived as the target of a social interaction that cannot be avoided.

Panel A)

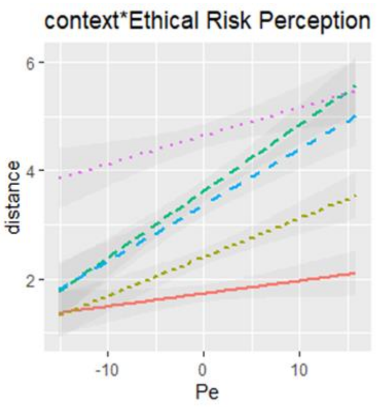

Panel B)

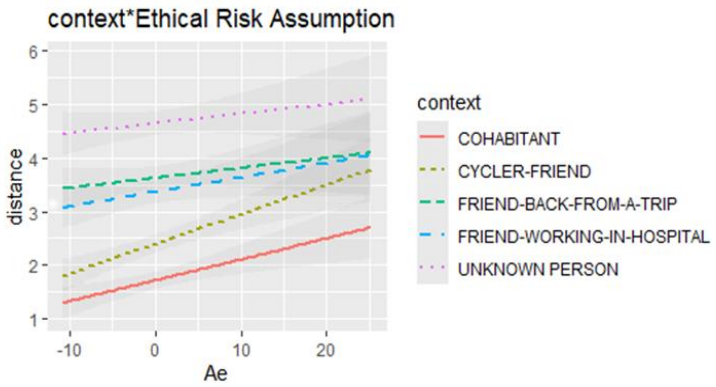

Supplementary-Figure 6) PANEL A) The graph shows the predicted values of the outcome variables. Shaded bands represent the confidence intervals (95%). The more participants showed high scores of Ethical Risk perception, the more they decided to keep a distance from the three categories of friends. PANEL B) The graph shows the predicted values of the outcome variables. Shaded bands represent the confidence intervals (95%). The more participants showed high scores of Ethical Risk Assumption, the more they decided to keep a distance from the Cycler Friend.

Recreational Risk Perception and Assumption of the Domain-Specific Risk-Taking (DOSPERT) Interpersonal distance

The analysis included as continuous predictors the Recreational Risk Perception and Assumption of the Domain-Specific Risk-Taking (DOSPERT) scale and as categorical predictors the Context (FRIEND-WORKING-IN-HOSPITAL, FRIEND-BACK-FROM-A-TRIP, CYCLER-FRIEND, UNKNOWN PERSON, COHABITANT), participants and color combinations were kept as random intercepts. The model yielded a significant main effect of the Context (FRIEND-WORKING-IN-HOSPITAL, FRIEND-BACK-FROM-A-TRIP, CYCLER-FRIEND, UNKNOWN PERSON, COHABITANT), (F(4,1727.115)=235.2435, p<.00001) the continuous predictor Recreational Risk Perception (F(1,55.587)=4.7820, p=.03298) and of the continuous predictor Recreational Risk Assumption (F(1,56.357)=6.1175, p=.01642). The two-way interaction between the continuous predictor Recreational Risk Perception and the categorical predictor Context was significant (F(4,1727.003)=10.2581, p<.00001) and the two-way interaction between the continuous predictor Recreational Risk Assumption and the categorical predictor Context was significant (F(4,1727.005)=2.8150, p=.02410). Simple slope analysis showed that the slopes of the FRIEND-BACK-FROM-A-TRIP [LCI 0.0434 – UCI 0.1746] and of the UNKNOWN PERSON [LCI 0.0465- UCI 0.1779] were significantly different from zero as a function of the Recreational Perception Risk. The pairwise difference between the simple slopes of the FRIEND-BACK-FROM-A-TRIP and the CYCLER-FRIEND (estimate = 0.06189 , SE =0.0193, t(1727)=3.202 p=.0121) were significant. The pairwise difference between the simple slopes of the FRIEND-BACK-FROM-A-TRIP and the FRIEND-WORKING-IN-HOSPITAL as a function of the Recreational Risk Perception was significant (estimate=0.05662 , SE 0.0193, t(1727)=2.929 p=.0284) and the pairwise difference between the simple slopes of the FRIEND-BACK-FROM-A-TRIP and the COHABITANT (estimate=0.09901, SE =0.0193, t(1727)=5.122 p<.0001) was significant. The pairwise difference between the simple slopes of the CYCLER-FRIEND and the UNKNOWN PERSON as a function of the Recreational Risk Perception was significant (estimate= -0.06515, SE =0.0193, t(1727)=-3.37 p=.0069) and the pairwise difference between the simple slopes of the COHABITANT and the UNKNOWN PERSON (estimate= -10.227, SE =0.0193, t(1727)=-5.290 p<.0001) was significant Fig.7).

The Recreational Risk perception subscale has an impact on the friend back from the trip and the unknown person, which, as we have previously speculated, can be considered as out-group members: a person with whom we have never interacted before and a person which has just been outside the country. The more participants scored high on the Recreational Risk perception subscale, the more they kept distance from the friend back from the trip and the unknown person. The recreational domain is related to extreme or outdoor activities. Generally, many items overlap with items on the Sensation Seeking Scale[51], and risks are about personal health and safety issues. Thus, the two out-group members (the unknown person and the friend back from the trip) are those impacted by this subscale.

Simple slope analysis showed that the slopes of the FRIEND-BACK-FROM-A-TRIP [LCI 0.000119 – UCI 0.0881] of CYCLER-FRIEND the [LCI 0.027104-UCI 0.1151] and of the COHABITANT [LCI 0.0162 - UCI 0.1042] were significantly different from zero as a function of the Recreational Assumption Risk. The pairwise difference between the simple slopes of the CYCLER-FRIEND and of the UNKNOWN PERSON as a function of the Recreational Risk Assumption was significant (estimate=0.03512, SE =0.0128, t(1727)=2.740 p=.0487) Figure 7). Results indicate that the more participants scored high in the Recreational Risk Assumption, the more they kept distance from the cohabitant and cycler friend and from the friend back from the trip. The pattern of results resembles the one in the Health/Safety Risk Assumption, with the difference that here, Recreational Risk Assumption impacts not only on the cycler friend and the cohabitant, but also on the friend back from the trip. As we already claimed, in the recreational domain risks are about personal health and safety issues, additionally many items overlap with items on the Sensation Seeking Scale[51], which might explain why the Recreational Risk Assumption impacts also on a social target perceived dangerous compared to the other who is the friend back from the trip.

Panel A) Panel B)

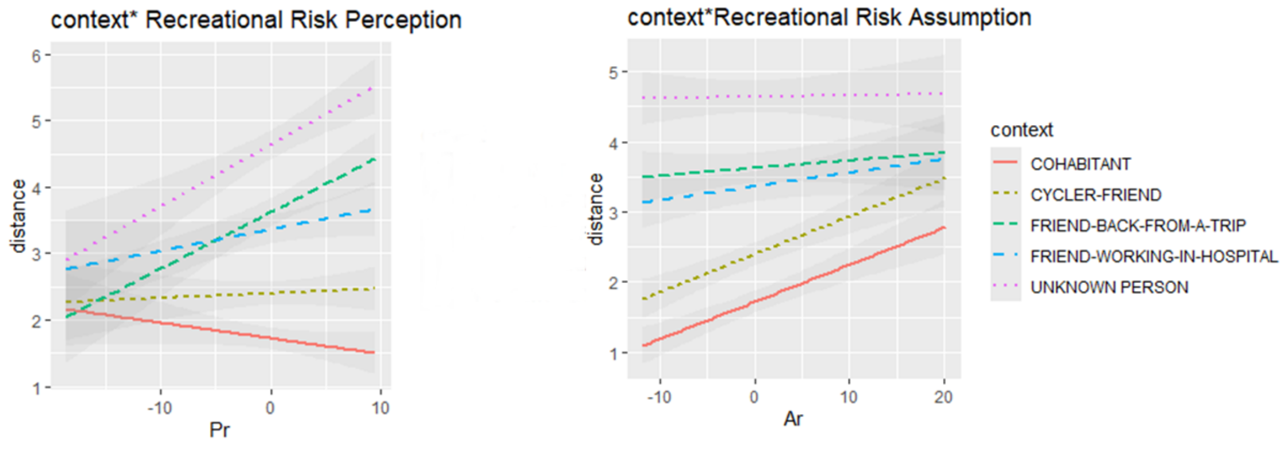

Supplementary-Figure 7) Panel A) The graph shows the predicted values of the outcome variables. Shaded bands represent the confidence intervals (95%). The more participants showed high scores of Recreational Risk perception, the more they decided to keep a distance from the Unknown person and the Friend back from a trip. Panel B) The graph shows the predicted values of the outcome variables. Shaded bands represent the confidence intervals (95%). The more participants showed high scores of Realistic Threat, the more they decided to keep a distance from the Cohabitant the Cycler friend and the Friend back from the trip.

Financial Investment Risk Perception of the Domain-Specific Risk-Taking (DOSPERT) and Interpersonal distance

The Financial Risk Perception and Assumption can be further decomposed into gambling and investment measures. In our model, the continuous predictors were the Financial Investment Risk Perception and the Assumption of the Domain-Specific Risk-Taking (DOSPERT) scale, and the categorical predictors were the Context (FRIEND-WORKING-IN-HOSPITAL, FRIEND-BACK-FROM-A-TRIP, CYCLER-FRIEND, UNKNOWN PERSON, COHABITANT). Participants and color combinations were kept as random intercepts. The model yielded a significant main effect of the Context (FRIEND-WORKING-IN-HOSPITAL, FRIEND-BACK-FROM-A-TRIP, CYCLER-FRIEND, UNKNOWN PERSON, COHABITANT), (F(4,1727.006)=228.7665, p=.00001), of the continuous predictor Financial Investment Risk Perception (F(1,55.930)= 5.8256, p=.0190946). The two-way interaction between the categorical predictor Context and the continuous predictor Financial Investment Risk Perception was significant (F(4,1727.003)=4.9398, p=.0005832). Simple slope analysis showed that the slopes of the FRIEND-BACK-FROM-A-TRIP [LCI 0.03525 – UCI 0.203] and the FRIEND-WORKING-IN-HOSPITAL, [LCI 0.03251- UCI 0.200] were significantly different from zero as a function of Financial Investment Risk Perception. Simple slope analysis showed that the slopes of the five levels of the Contexts were not significantly different from zero as a function of Financial Investment Risk Assumption, Figure 8). The covariate Financial Investment Risk Perception impacts the preferred interpersonal distance from the friend back from a trip and the friend working in hospital. It can be speculated that these categories of friends are those considered more dangerous compared with the cycler friend, whereas again, the cohabitant and the unknown person might be perceived outside the sphere of the social distancing responsibility. The Financial Investment Risk Perception measures the perception of the risk/benefit to investing money in a stock with moderate risk. Such perception might be translated to the perception of the risk/benefit when being closer to potentially dangerous friends.

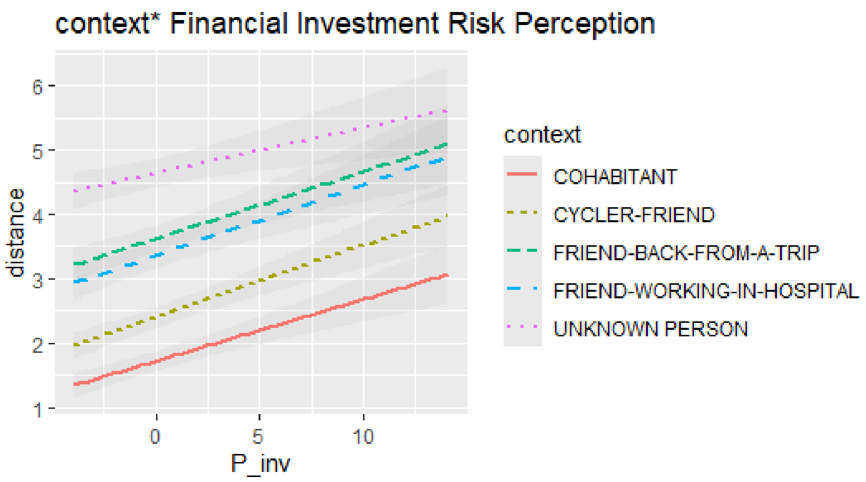

Supplementary- Figure 8) The graph shows the predicted values of the outcome variables. Shaded bands represent the confidence intervals (95%). The more participants showed high scores of Financial Investment Risk perception, the more they decided to keep a distance from the Friend back from a trip and the Friend working in hospital.

Financial Gambling Risk Perception and Assumption of the Domain-Specific Risk-Taking (DOSPERT) and Interpersonal distance

We then created a model with the Financial Gambling Risk Perception and Assumption of the Domain-Specific Risk-Taking (DOSPERT) scale as continuous predictors and the Context (FRIEND-WORKING-IN-HOSPITAL, FRIEND-BACK-FROM-A-TRIP, CYCLER-FRIEND, UNKNOWN PERSON, COHABITANT) as categorical predictors. Participants and color combinations were kept as random intercepts. The model yielded a significant main effect of the Context (FRIEND-WORKING-IN-HOSPITAL, FRIEND-BACK-FROM-A-TRIP, CYCLER-FRIEND, UNKNOWN PERSON, COHABITANT), ($F(4,1727.006)=232.3878$, $p<.00001$), of the continuous predictor Financial Gambling Risk Perception ($F(1,54.371)= 6.6083$, $p=.0129202$). The two-way interaction between the categorical predictor Context and the continuous predictor Financial Gambling Risk Perception was significant ($F(4,1727.005)=11.4618$, $p<.00001$). Simple slope analysis showed that the slopes of the FRIEND-BACK-FROM-A-TRIP [LCI 0.090507 – UCI 0.2676], of the FRIEND-WORKING-IN-HOSPITAL [LCI 0.008102- UCI 0.1852], and of the UNKNOWN PERSON [LCI 0.066046- UCI 0.2431] were significantly different from zero as a function of Financial Gambling Risk Perception. The pairwise difference between the simple slopes of the COHABITANT and the CYCLER-FRIEND as a function of the Financial Gambling Risk Perception (estimate=-0.08487, SE=0.286, $t(1727)=-2.968$ $p=0.0252$) was significant, as the pairwise difference between the simple slopes of the COHABITANT and of the FRIEND-BACK-FROM-A-TRIP (estimate=-0.17620, SE=0.286, $t(1727)=-6.163$ $p<0.0001$). Both the pairwise difference between the simple slopes of the COHABITANT and of the FRIEND-WORKING-IN-HOSPITAL (estimate=-0.09380, SE=0.286, $t(1727)=-3.281$ $p=0.0093$) and the pairwise difference between the simple slopes of the COHABITANT and of the UNKNOWN PERSON (estimate= -0.15175, SE=0.286, $t(1727)=-5.306$ $p<0.0001$) were significant. Finally, the pairwise difference between the simple slopes of the CYCLER-FRIEND and of the FRIEND-BACK-FROM-A-TRIP (estimate = -0.09133, SE=0.286, $t(1727)=-3.194$ $p=0.0124$) and the pairwise difference between the simple slopes of the FRIEND-BACK-FROM-A-TRIP and of the FRIEND-WORKING-IN-HOSPITAL (estimate = 0.08240, SE=0.286, $t(1727)=2.882$ $p=0.0326$) were significant Figure 9). The Financial Gambling Risk Perception impacts on the preferred interpersonal distance from the two more dangerous categories of friends and on the unknown person. The more participants scored high in the financial gambling risk perception, the more they kept a distance from these categories of people perceived as dangerous. We can speculate that the perception of the risk/benefit of a financial bet outcome, which can change the player's life, can also be spread to the perception of the health/sanitary risk exposure, expressed by the social distancing towards the risky social categories.

Simple slope analysis showed that the slopes of the FRIEND-BACK-FROM-A-TRIP [LCI 0.0743 – UCI 0.210] of the FRIEND-WORKING-IN-HOSPITAL [LCI 0.0702- UCI 0.205], of the UNKNOWN PERSON [LCI 0.0315 – UCI 0.167], of the COHABITANT [LCI 0.0380 – UCI 0.174], and of the CYCLER-FRIEND [LCI 0.0696- UCI 0.205], were significantly different from zero as a function of the Financial Gambling Risk Assumption Figure)9). These results are quite impressive and show that the Financial Gambling Risk Assumption impacts all the social categories proposed. In other words, scoring high to the Financial Gambling Risk Assumption means to take more distance from all the social categories. There is not any interpersonal distance immune from being impacted by the Financial Gambling Risk Assumption. It seems that the attitude to take the financial risk overlaps with taking the healthy/safety risk towards everybody.

Panel A) Panel B)

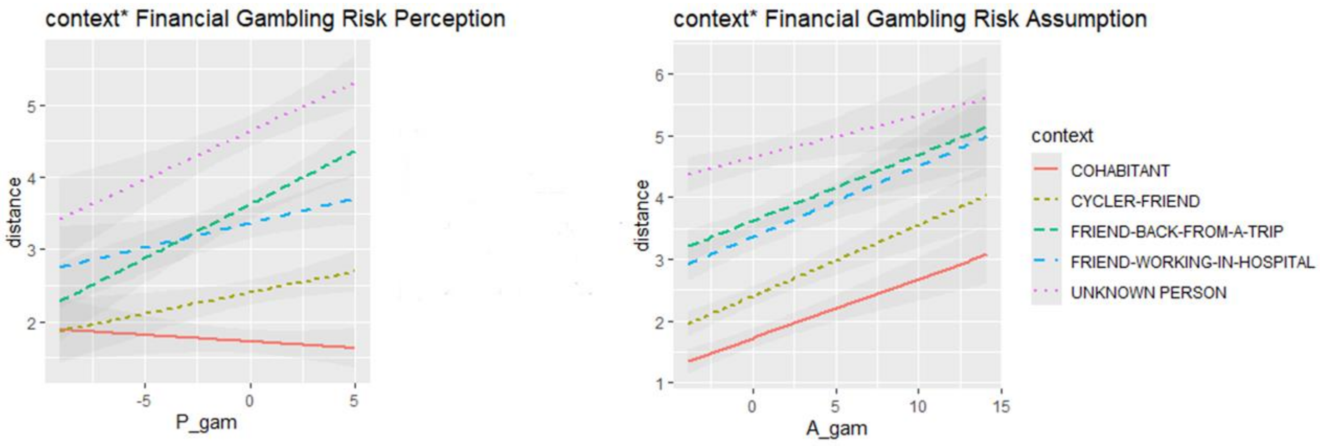

Supplementary- Figure 9) Panel A) The graph shows the predicted values of the outcome . Shaded bands represent the confidence intervals (95%). The more participants showed high scores of Financial Gambling Risk Perception, the more they decided to keep a distance from the Friend back from a trip, the Friend working in the Hospital and the Unknown person. Panel B) The graph shows the predicted values of the outcome variables. Shaded bands represent the confidence intervals (95%). The more participants showed high scores of Financial Gambling Risk Assumption, the more they decided to keep a distance from all the social categories.

Discussion

The ethical risk perception subscale of the Domain-Specific Risk-Taking (DOSPERT) [37] has the same pattern of the health/safety risk perception subscale. Experiencing the risk that our health is vulnerable to the infection impacts on the preferred interpersonal distance which participants decide to keep from friends and not from the cohabitant or the unknown person. The results suggest that being in a close relation with someone

induces the ethical conflict between the need to maintain the emotional bond with friends and the responsibility towards the community in respecting the social norms.

The social and recreational risk perception subscales measure the respondents' level assessment respectively of how risky each behavior is for social decisions (Disagreeing with an authority figure on a major issue, Speaking about an unpopular issue in a business meeting) or for outdoor recreational activities (Bungee jumping off a tall bridge, Going camping in the wilderness). The more people scored high on the social and recreational perception risk assumption, the more they kept a distance from the two social categories considered as more dangerous. These subscales impact only the interpersonal distance from the unknown person, and the friend just got off from an international flight. When the data were collected (from the second half of July to the end of August 2020), Europe more than Italy was hit hard by the first pandemic wave; people who came from outside the country were perceived as more exposed to the Covid-19 infection. Probably, the unknown person and the friend just got off from an international flight were the social categories perceived as more dangerous. The social risk perception subscale implies the courageous social exposure to life's events and others' disapproval. In contrast, the recreational risk perception subscale pertains to the courageous exposure to safety issues due to the extreme sport practicing. We can speculate that the social distancing from the social categories which were perceived as more dangerous was either affected by the awareness of "who" were the others (social risk perception subscale) and consequently by the conveyed threat by them (recreational risk perception subscale).

Finally, the financial investment risk perception subscale impacts the interpersonal distance with the friend just got off from an international flight and the friend working in a hospital, whereas the financial gambling risk perception modulates the interpersonal distance with the two above mentioned friends and also with the unknown person. The more people scored high on the above mentioned financial risk subscales, the more they kept a distance from the friends considered at risk of infection and the unknown person. These results can be interpreted by considering that either the perception of the risk/benefit to investing money in a stock with moderate risk (Financial Investment Risk Perception), either of the risk/benefit of a financial bet outcome, which can change the player's life (Financial Gambling Risk Perception), can be spread to the perception of a health/sanitary exposure expressed by the social distancing towards the riskiest social categories.

The risk assumption subscales of the Domain-Specific Risk-Taking DOSPERT[37] measure the respondents' self-reported likelihood of engaging in risky behaviors as "risk-taking." It is interesting to remark the fact that risk perception and risk-taking scales are negatively correlated. Thus it makes sense that the scales differently impact the social categories. The results indicate that the healthy/safety risk assumption impacts the interpersonal distance with the cohabitant and the friend back from the cycling ride, whereas the ethical risk influences only the interpersonal distance with the latter. The more participants scored high on the

healthy/safety risk assumption, the more they kept a distance from the cohabitant and the friend back from the cycling ride, whereas the more scored high on the ethical risk assumption, the more they stayed far from the friend back from the cycling ride. It might be speculated that while the interpersonal distance from the social categories depicted as "more dangerous" is regulated by the risk perception of different behaviors, the interpersonal distance from the social categories depicted as "less dangerous" is regulated by the risk-taking of the same behaviors. Indeed, the impact of the risk perception on social targets prevents the risk assumption's impact on the same social targets. Finally, the recreational risk assumption subscale modulates the interpersonal distance with the friend back from the cycling ride, the cohabitant, and also the unknown person. And the financial gambling risk assumption subscale shows modulation of the interpersonal distance with all the selected five social targets. We can notice that these last two subscales involve a dimension of sensation seeking, which might lead to overlapping between the impact of risk perception and the impact of the risk assumption on the same social targets, see Table 4 for a general synthesis).

Integrated Covid scale	DOSPERT Health/Safety scale		DOSPERT Ethical scale		DOSPERT Social scale		DOSPERT Recreational scale		DOSPERT Financial Investment scale		DOSPERT Financial Gambling scale	
Realistic threat	Risk perc	Risk ass	Risk perc	Risk ass	Risk perc	Risk ass	Risk perc	Risk ass	Risk perc	Risk ass	Risk perc	Risk ass
FrHosp	FrHosp	FrCycl	FrHosp	FrCycl	FrTrip	/	FrTrip	FrCycl	FrHosp	/	FrHosp	FrHosp
FrTrip	FrTrip	Cohab	FrTrip	/	Unkn	/	Unkn	Cohab	FrTrip	/	FrTrip	FrTrip
FrCycl	FrCycl	/	FrCycl	/	/	/	/	FrTrip	/	/	Unkn	FrCycl
/	/	/	/	/	/	/	/	/	/	/	/	Cohab
/	/	/	/	/	/	/	/	/	/	/	/	Unkn

Supplementary-Table 4) A summary depicting the impact of each covariate on the different contexts.
